# Quality of life, stigma, and mental health, in individuals affected by leprosy: a cross-sectional study

**DOI:** 10.1093/trstmh/trag064

**Published:** 2026-05-28

**Authors:** Temesgen K Chala, Emily S Garsleitner, Guenter Froeschl, Esayas K Gudina, Kristina Adorjan

**Affiliations:** CIH^LMU^ Center for International Health, Ludwig-Maximilians-Universität München, Munich 80802, Germany; Department of Health Policy and Management, Jimma University, Jimma, Oromia 378, Ethiopia; CIH^LMU^ Center for International Health, Ludwig-Maximilians-Universität München, Munich 80802, Germany; Institute of Infectious Disease and Tropical Medicine, University Hospital (LMU), Munich 80802, Germany; CIH^LMU^ Center for International Health, Ludwig-Maximilians-Universität München, Munich 80802, Germany; Institute of Infectious Disease and Tropical Medicine, University Hospital (LMU), Munich 80802, Germany; Department of Internal Medicine, Jimma University, Jimma 378, Ethiopia; Clinical Trial Unit, Jimma University, Jimma 378, Ethiopia; CIH^LMU^ Center for International Health, Ludwig-Maximilians-Universität München, Munich 80802, Germany; Department of Psychiatry and Psychotherapy, University of Bern, Bern 3000, Switzerland; Institute of Psychiatric Phenomics and Genomics (IPPG), LMU University Hospital Munich, Munich 80802, Germany; Multidisciplinary Center for Infectious Diseases (MCID), University of Bern, Bern 3000, Switzerland

**Keywords:** Ethiopia, leprosy, mental health, QoL, stigma

## Abstract

**Background:**

Leprosy rarely causes death but leads to disfigurement, stigma, and disability, resulting in socioeconomic consequences. Understanding these inter-related factors is essential for developing effective interventions. This study aimed to assess QoL and mental health among individuals affected by leprosy and to examine identify QoL predictors.

**Methods:**

A cross-sectional study was conducted in urban leprosy rehabilitation centres in Ethiopia. A total of 370 individuals were enrolled. Correlation and multivariable regression analyses were used to examine associations and identify predictors of QoL.

**Results:**

Participants were predominantly male (54.1%) and aged ≥61 years (42.8%). Most had no education (57.3%), while only 4.3% had secondary education or above; one-half were married. Poor living conditions were reported in 84.05% of participants, while delayed diagnosis (≥2 years after symptom onset) was observed in 53% of cases. The median social relationship QoL score was 7 (IQR 5–9), while that for overall QoL was 61 (IQR 56–68). Median sleep score was 8 (IQR 6–11). Stigma correlated positively with anxiety (r = 0.206) and depression (r = 0.269) and negatively with QoL (r = −0.251). Anxiety and depression were strongly correlated (r = 0.794, *P* < .001).

**Conclusions:**

Stigma, psychological distress and poor sleep impair QoL among people affected by leprosy, highlighting the need for multidisciplinary interventions.

## Introduction

Leprosy is an infectious disease that has existed since ancient times.^1^ It causes skin lesions, disfigurements, and damage to peripheral nerves in different parts of the body. These effects often lead to loss of sensation, which can result in secondary infections. The introduction of multidrug therapy (MDT), combined with national leprosy-elimination programmes, has resulted in progress towards controlling leprosy in the public health systems of endemic countries.^2^ Despite these successes, deep-rooted misbeliefs of contagion and impurity persist in leprosy-endemic settings,^3^ impeding disease-elimination efforts.

Leprosy is often associated with stigma and discrimination, resulting in serious social, psychological, and economic effects.^4^ Deep-rooted religious, cultural, and moral beliefs sustain discrimination and delay diagnosis and treatment, raising the risk of serious disabilities.^5^ Stigma remains one of the most enduring consequences of leprosy. It functions in several ways, including experienced, expected, internalized, and enacted stigma, all of which influence the social and psychological consequences for affected individuals and their families.

The stigmatization of leprosy has deep roots.^6^ In ancient civilizations such as India, China, Egypt, and Mesopotamia, leprosy was viewed as a sign of divine punishment or moral failure, a mark of spiritual impurity.^1^ Furthermore, stigma is a documented barrier to health-seeking behaviour, engagement in care, and adherence to treatment, and in the case of leprosy,^7^ it has become more destructive and enduring than the disease itself, shaping lives through exclusion, fear, and silence across generations.

Stigma reduces social engagement, increases feelings of shame, and contributes to a decline in self-worth. As a result, individuals affected by leprosy experience lower quality of life (QoL). The WHO defines QoL as the ‘individual’s perception of their position in life in the context of the culture and value systems in which they live and in relation to their goals, expectations, standards and concerns’.^8^

Stigma and reduced QoL often lead to mental health disorders.^9^ Evidence shows that people affected by leprosy experience much higher rates of depression and anxiety compared with the general population.^9^ Moreover, the physical, psychological, and social effects of leprosy contribute to sleep problems. Insomnia, exhaustion, irritability, and poor daytime functioning, often resulting from neuropathic pain, nerve damage, and psychological distress, interfere with normal sleep patterns.^10,11^

In Ethiopia, there is a paucity of data regarding the QoL and mental health status of individuals affected by leprosy. Therefore, understanding both the QoL and mental health status of this population is essential for generating data that can guide targeted interventions and promote social reintegration. Thus, this study aims to (i) determine the perceived stigma, general QoL, mental health status, and sleep quality of individuals affected by leprosy in Ethiopia; and (ii) evaluate the correlation between demographic factors, stigma scores, and leprosy-related clinical factors on one hand, and QoL and mental health on the other.

## Materials and methods

### Study setting and design

This study is part of a larger project aimed at improving community awareness, reducing stigma, examining the situation of patients and healthcare workers, and describing care pathways among leprosy-affected individuals. This study was located in Ethiopia, one of the countries located in the Horn of Africa. The country is the second most populous country on the continent, with an estimated population of 130 million.

The country has a long-standing history of addressing leprosy and is home to several specialized centres dedicated to the diagnosis, treatment, and rehabilitation of individuals affected by the disease. There are six primary leprosy care centres across the country: Relief Center Bisidimo, Dessie Boru Meda, Shashemene, Gambo, Bushulo, and the All-Africa Leprosy Tuberculosis and Rehabilitation Training Center (ALERT), which is internationally recognized for its role in leprosy research and care.

In addition to these government-supported facilities, leprosy care is also provided by institutions established through partnerships with faith-based and non-governmental organizations. Notably, the Catholic Church and the Ethiopian National Association of People Affected by Leprosy (ENAPAL), in collaboration with the German Leprosy and TB Relief Association (GLRA), contribute significantly to service delivery, advocacy, and community-based rehabilitation efforts throughout the country. The Jimma Leprosy Rehabilitation Center is one of the facilities established by faith-based organizations. These collaborative networks have helped provide comprehensive care and support for individuals affected by leprosy across Ethiopia. This study was conducted at selected leprosy rehabilitation centres, namely, Jimma, ALERT, and Shashemene. A facility-based cross-sectional study was conducted from 16 February to 15 December 2024.

### Selection of study participants

The study participants consisted of individuals affected by leprosy who were either receiving treatment or rehabilitation services at the selected centres. Because the population was finite, we applied Cochran’s equation together with a population correction formula to calculate the sample size, resulting in a target sample size of 385 individuals. The total sample size calculated was then proportionally allocated to the study sites based on the site’s annual leprosy report (unpublished reports from 2023 were considered from each rehabilitation site), which were 232 in Jimma, 330 in ALERT, and 204 in Shashemene in 2021. Study participants were selected using a systematic sampling interval from registrations. Finally, a sampling interval of two for Jimma and six each for Shashemene and ALERT was applied.

### Inclusion criteria

Individuals who were registered as patients with leprosy at the rehabilitation centres (both those who completed and those who are currently receiving MDT), aged ≥18 years, were invited to participate in this study. Of those individuals, only the ones who provided voluntary informed consent were included in this study. On the contrary, individuals with severe disabilities, either those receiving care (e.g. reconstructive surgery) or those confined to bed at home, were excluded.

The questionnaire was administered face-to-face. Variables for leprosy-related clinical data collection were adapted from previous literature.^12^ The other part of the questionnaire included the outcome variables, which are standardized instruments^13^ we used without modifications; in addition, a questionnaire was developed to collect sociodemographic and clinical data on leprosy-related variables. The data collection tool was initially developed in English, then translated into the local languages, Afan Oromo and Amharic. The questionnaire was back-translated to ensure consistency. Finally, the questionnaire was deployed using Research Electronic Data Capture (REDcap) to enable efficient and secure digital data collection. A total of six data collectors were recruited and trained for 2 days. A pilot test was conducted to ensure clarity and data quality.

### Outcome assessment

#### Quality of life (primary outcome)

##### WHO Quality of Life—BREF 

The WHO Quality of Life—BREF (WHOQOL-BREF) is a widely used, brief QoL assessment tool derived from the longer WHOQOL-100 instrument. It consists of 26 items covering four domains: Physical Health, Psychological Health, Social Relationships, and Environment. Each domain represents a distinct facet of QoL, and the tool is designed to be culturally sensitive and applicable across diverse populations.^14^

##### Scoring and transformation

Questions 4 and 26 of the WHOQOL-BREF were reverse-scored, as higher scores on these items indicate poorer QoL, contrary to the overall scoring direction. Raw domain scores were calculated by averaging the responses to items within each domain. These raw scores were then linearly transformed to a 0–100 scale, with higher scores indicating better QoL and lower scores indicating poorer QoL. Finally, we calculated the global QoL score by summing all domains and the overall QoL and general health questions.

##### Pittsburgh Sleep Quality Index 

Sleep quality over the past month was measured using the Pittsburgh Sleep Quality Index (PSQI), which produces seven component scores (subjective sleep quality, sleep latency, sleep duration, habitual sleep efficiency, sleep disturbances, use of sleep medication, and daytime dysfunction). Each component was scored 0 = no difficulty/not during the past month, 1 = mild difficulty/less than once a week, 2 = moderate difficulty/once or twice a week, or 3 = severe difficulty/three or more times a week. Component scores were summed to yield a global PSQI score from 0 to 21, where higher scores reflect poorer sleep quality. The PSQI has been psychometrically validated for assessing sleep quality.^15^

#### Mental health disorders (secondary outcome)

##### Patient Health Questionnaire-9 

Depressive symptoms were assessed using the Patient Health Questionnaire-9 (PHQ-9), with each item rated on a scale from 0 (not at all) to 3 (nearly every day). Total scores ranged from 0 to 27, interpreted according to standard cut-offs: 0–4 = minimal depression, 5–9 = mild depression, 10–14 = moderate depression, 15–19 = moderately severe depression, and 20–27 = severe depression. The PHQ-9 has been validated for assessing depression.^16^

##### Generalized Anxiety Disorder-7

Anxiety was assessed using the Generalized Anxiety Disorder-7 (GAD-7) screener. Item scores were summed to yield a total score from 0 to 21, with interpretation as follows: 0–4 = minimal anxiety, 5–9 = mild anxiety, 10–14 = moderate anxiety, and 15–21 severe anxiety. The GAD-7 has been validated and demonstrates internal consistency.^17^

#### Factors contributing to quality of life and mental health

##### Stigma Assessment and Reduction Instrument

Leprosy-related stigma was measured with the 37-item Stigma Assessment and Reduction Instrument (SARI), covering eight subtypes: experienced/enacted, anticipated, internalized, self-stigma, alienation, stereotyping, discrimination, and social withdrawal. Each statement was rated on a five-point Likert scale (1 = absolutely yes, 2 = rather yes, 3 = undecided, 4 = rather no, 5 = absolutely no). The items were reverse-coded so that higher numerical values indicate greater stigma. The SARI was specifically validated among leprosy-affected individuals and has demonstrated internal consistency and the ability to capture multiple stigma dimensions.^18^

##### Leprosy-specific and sociodemographic variables

In addition to the outcome variables, sociodemographic and leprosy-related variables were collected. Individuals were classified according to the WHO Expert Committee on Leprosy grading system upon registration at the rehabilitation centres, and this information was obtained from the registration books maintained at the centres.^19^

###### Grouping variable

Participants were divided based on their WHO leprosy grading into two groups: advanced (grade 1 and 2 disability) and non-advanced (grade 0 disability) (G0D). Using the standardized WHO grading facilitated easier comparisons with other studies. The goal of this grouping was to compare socioeconomic variables between the groups.

#### Data management

Data collected through REDcap were synchronized with the Jimma University server and were extracted in Excel format. Data cleaning and preliminary analysis were performed using Microsoft Excel. The dataset was then exported to Stata version 17 (StataCorp, USA) for statistical analysis. Descriptive statistics, including median, interquartile range, frequency, and percentage, were used to summarize the data. Bivariate regression was applied to identify candidate variables using a significance level of *P* < .25. These variables were subsequently included in a multivariable regression model to determine independent predictors of the outcome variables.

Residual normality was assessed using skewness and kurtosis, where values between −1 and 1 indicated a normal distribution. The Breusch–Pagan/Cook–Weisberg test was used to assess heteroscedasticity in the regression model, with the assumption of normally distributed error terms. The test was conducted using the fitted values of the outcome variables. The test produced a chi-square statistic of 0.05 with *P* = .8. Hence, we failed to reject the null hypothesis of constant variance.

The Breusch–Pagan/Cook–Weisberg test indicated no evidence of heteroscedasticity, confirming that the assumption of homoscedasticity holds in the model. Potential outliers were assessed using Cook’s distance, with none identified as influential based on the ±1 threshold. Multicollinearity among independent variables was evaluated using the variance inflation factor (VIF). Initially, global PHQ score, global GAD-7 score, anxiety levels, depression levels, age, and age group exhibited high multicollinearity, with VIFs >10, suggesting strong correlations among these variables.

To address this, the categorical variables (anxiety levels, depression levels, and age group) were removed from the correlated set (Supplementary file 1). Following this adjustment, the mean VIF decreased to 1.86, indicating that multicollinearity was no longer a concern. Statistical significance of independent predictors was determined at *P* < .05 and a 95% confidence level. We used the STrengthening the Reporting of OBservational studies in Epidemiology (STROBE) reporting guideline^20^ to draft this manuscript, and the STROBE reporting checklist when editing.

#### Declaration on the use of AI

After a personal compilation of existing evidence in the discussion section, Chat gpt ai 4.0 has been used to complement the compiled evidence. It has also been used for English language correction after the full manual writing of this thesis.

## Results

### Sociodemographic characteristics

A slightly higher proportion of females (68.2%) were at an advanced disease stage compared with males (63.5%). Being at an a dvanced disease stage was most common among participants aged ≥61 years, affecting 71.5% in this age group, which was significant (*P* = .022). Being at an advanced disease stage was more common in widowed participants (72.8%) and married participants (68.3%). There were no notable differences based on family size, education level, income group, or family history of leprosy (Tables 1–7).

**Table 1:** Association between disease stage and age group of the study participants (*n* = 370).

	Age group (years)				
Disease stage	18–30	31–45	46–60	≥61	Total
Non-advanced	23	26	33	45	127
	53.5%	36.6%	33.7%	28.5%	34.3%
Advanced	20	45	65	113	243
	46.5%	63.4%	66.3%	71.5%	65.7%
Total	43	71	98	158	370
	100.0%	100.0%	100.0%	100.0%	100.0%
Pearson chi^2^(3)	9.5830; *P* = .022				

**Table 2:** Association between disease stage and gender of the study participants (*n* = 370).

	Gender		
Disease stage	Female	Male	Total
Non-advanced	54	73	127
	31.8%	36.5%	34.3%
Advanced	116	127	243
	68.2%	63.5%	65.7%
Total	170	200	370
	100.0%	100.0%	100.0%
Pearson chi^2^(1) = 0.9140, *P* = .339			

**Table 3:** Association between disease stage and income group of the study participants (*n* = 370).

	Income group						
Disease stage	Extremely poor	Moderate	Near poor	Low income	Middle income	Upper income	Total
Non-advanced	30	23	24	33	13	4	127
	31.3%	37.1%	34.8%	39.3%	33.3%	20.0%	34.3%
Advanced	66	39	45	51	26	16	243
	68.8%	62.9%	65.2%	60.7%	66.7%	80.0%	65.7%
Total	96	62	69	84	39	20	370
	100.0%	100.0%	100.0%	100.0%	100.0%	100.0%	100.0%
Pearson chi^2^(5) = 3.3750, *P* = .642							

**Table 4:** Association between disease stage and family size among the study participants (*n* = 370).

	Family size				
Disease stage	0–3	4–6	7–10	≥10	Total
Non-advanced	70	37	18	2	127
	32.9%	32.7%	43.9%	66.7%	34.3%
Advanced	143	76	23	1	243
	67.1%	67.3%	56.1%	33.3%	65.7%
Total	213	113	41	3	370
	100.00	100.00	100.00	100.00	100.00
Pearson chi^2^(3) = 3.3874, *P* = .336					

**Table 5:** Association between disease stage and family history of the study participants (*n* = 370).

	Family history of leprosy		
Disease stage	No	Yes	Total
Non-advanced	124	3	127
	34.2%	42.9%	34.3%
Advanced	239	4	243
	65.8%	57.1%	65.7%
Total	363	7	370
	100.0%	100.0%	100.0%
Pearson chi^2^(1) = 0.2304, *P* = .631			

**Table 6:** Association between disease stage and educational status of the study participants (*n* = 370).

	Educational status			
Disease stage	No formal	Primary	Secondary	Total
Non-advanced	71	49	7	127
	33.5%	34.5%	43.8%	34.3%
Advanced	141	93	9	243
	66.5%	65.5%	56.3%	65.7%
Total	212	142	16	370
	100.0%	100.0%	100.0%	100.0%
Pearson chi^2^(2) = 0.6981, *P* = .705				

**Table 7:** Association between disease stage and marital status of the study participants (*n* = 370).

	Marital status				
Disease stage	Married	Separated	Single	Widow/widower	Total
Non-advanced	59	15	31	22	127
	31.72%	31.9%	55.4%	27.2%	34.3%
Advanced	127	32	25	59	243
	68.3%	68.1%	44.6%	72.8%	65.7%
Total	186	47	56	81	370
	100.0%	100.0%	100.0%	100.0%	100.0%
Pearson chi^2^(3) = 13.5140, *P* = .004					

### Clinical characteristics

Over two-thirds (65.1%) had visible deformities. Among the respondents, 41.9% were in the Grade 2 disability (G2D) group, and 23.8% were in the Grade 1 disability (G1D) group. Among the participants, 15.4% had at least one comorbidity. Hypertension was the most common (9.7%), followed by diabetes mellitus (4.6%) and tuberculosis (1.1%).

### Quality of life

The median global QoL score was 61 (IQR 56–68). Participants at a non-advanced disease stage had better global QoL than those at an advanced disease stage. The WHOQOL-BREF domains, namely, environmental (median = 17.5, IQR 15–20), psychological (median = 17, IQR 15–19), and physical health (median = 16, IQR 13–19) had the highest scores, while the social relationships domain had the lowest (median = 7, IQR 5–9). Higher education, younger age, and an absence of depression were associated with better QoL, especially in the environment, psychological health, and physical health domains.

### WHO BREF domains by gender

Males reported slightly higher median scores than females in the environment (18.07 vs. 17.50), psychological health (17.09 vs. 16.91), and physical health (16.05 vs. 15.88) domains, while the social relationships domain remained similarly low for both genders (Supplementary file 2).

### WHO BREF domains and leprosy grade

Environment and psychological health scores were slightly lower in individuals with G2D compared with those with lower grades. Physical health declined with increasing disability grade. The social aspect of the disease, however, is affected irrespective of the grade of leprosy (Figure 1).

**Figure 1:**
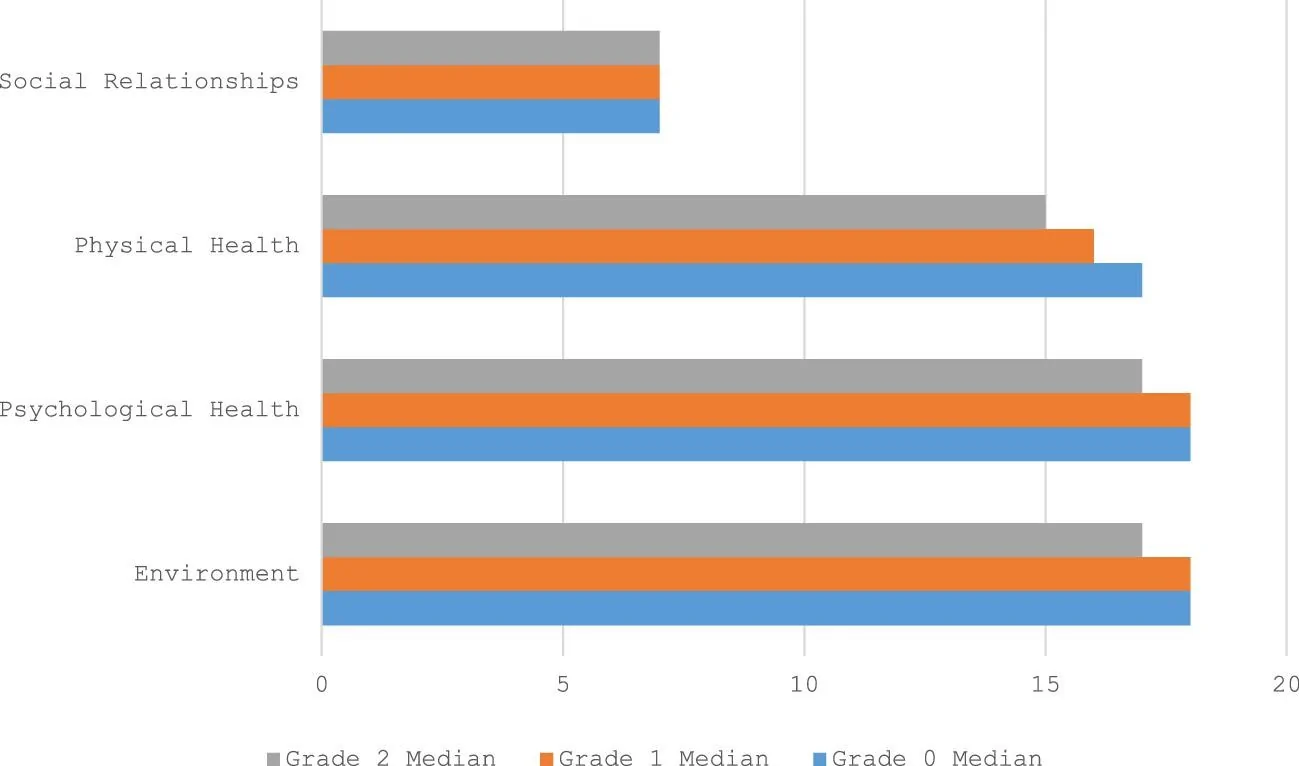
WHO BREF domains across different grades of leprosy (n=370).

### Quality of life, sleep quality, mental health, and stigma

The median QoL score was 61 (IQR 56–68). Sleep quality was poor among most participants, with a median PSQI score of 8 (IQR 6–11). Both PHQ-9 and GAD-7 median scores were 5, representing mild depression and anxiety, respectively. The prevalence of stigma was 26.2% (*n* = 97). Of these, 63.9% were at an advanced disease stage.

### Correlations between stigma, sleep, QoL, and mental health

Stigma (SARI score) was positively correlated with anxiety (r = 0.206, *P* < .05) and depression (r = 0.269, *P* < .05) and negatively correlated with QoL domains: global QoL (r = -0.251), environmental (r = -0.222), psychological (r = -0.273), and social relationships (r = -0.164) (all *P* < .05). Poor sleep (PSQI score) was positively correlated with depression (r = 0.201) and anxiety (r = 0.144) (*P* < .05). Depression and anxiety were also significantly correlated (r = 0.794, *P* < .001). QoL domains were strongly interrelated, with the strongest correlation between the physical health domain and global QoL (r = 0.723, *P* < .001) (Table 8).

**Table 8:** Spearman’s rank correlation among study participants (*n* = 370).

	Category of stigma (*ρ =* Spearman’s rho; *P = P* value)								
Factor	Experienced *(ρ, P)*	Internalized *(ρ, P)*	Anticipated *(ρ, P)*	Self *(ρ, P)*	Alienation *(ρ, P)*	Stereotype *(ρ, P)*	Discrimination *(ρ, P)*	Social withdrawal *(ρ, P)*	Stigma total *(ρ, P)*
Age	0.125, .0162*	0.040, .4439	0.015, .7800	0.124, .0167*	0.007, .9016	0.007, .9016	0.0430, .4096	−0.0319, .5408	0.093 .0736
Income	−0.297, .0000*	−0.184, .0004*	−0.205, .0001*	−0.197, .0001*	−0.262, .0000*	−0.325, .0000*	−0.3186, .0000*	−0.2964, .0000*	−0.3480 .0000*
Environment domain	−0.229, .0000*	−0.262, .0000*	−0.032, .5432	−0.042, .4161	−0.084, .1056	−0.117, .0246*	−0.1181, .0231*	−0.1264, .0150*	−0.1168 .0246*
Physical domain	−0.081, .1194	−0.069, .1850	0.100, .0544	0.016, .7592	−0.033, .5235	−0.077, .1377	0.0019, .9709	−0.0156, .7648	−0.0683 .1897
Psychological domain	−0.275, .0000*	−0.259, .0000*	−0.075, .1487	−0.134, .0097*	−0.136, .0086*	−0.141, .0068*	−0.0646, .2151	−0.1089, .0362*	−0.2760 .0000*
Social relationships domain	−0.178, .0006*	−0.179, .0005*	0.063, .2276	0.034, .5100	−0.072, .1684	−0.1663 .0013*	−0.0398, .4449	−0.0928, .0747	−0.1614 .0018*
PSQI	0.152, .0034*	0.126, .0155*	0.086, .0998	0.145, .0054*	0.114, .0285*	0.1268 .0147*	0.1014, .0514	0.0288, .5808	0.158 .0022*
PHQ-9	0.199, .0001*	0.242, .0000*	0.114, .0285*	0.138, .0081*	0.181, .0005*	*0.2095* .0000*	0.0710, .1732	0.1518, .0034*	0.250 .0000*
GAD-7	0.185, .0004*	0.221, .0000*	0.061, .2443	0.049, .3480	0.121, .0197*	0.1353 .0092*	0.0387, .4576	*0.1120* .0313*	*0.1973* .0001*

*Significant at *P* value ≤ 0.05.

### Predictors of global quality of life

The linear regression model showed a strong overall fit, thereby explaining a very high proportion of variance in global QoL (R² = 0.977, *P* < .001). Participants who completed at least primary school reported significantly higher QoL scores (β = 0.487, *P* = .015) than those with no formal education. Those with smaller families (≤3 members) had lower QoL scores by 0.621 units (β = −0.621, *P* = .035). All WHO BREF domain scores were strong positive predictors of global QoL: environment (β = 1.048, *P* < .001), physical health (β = 1.038, *P* < .001), social relationships (β = 1.070, *P* < .001), and psychological health (β = 1.059, *P* < .001). By contrast, moderately severe depression was significantly associated with lower QoL (β = −1.786, *P* = .003) (Supplementary file 3).

### Predictors for WHO BREF domains

In the physical domain, physical health was positively associated with environment, social relationships, and supernatural beliefs (*P* ≤ .05). In the psychological domain, the key factors affecting psychological health included age, anxiety, depression severity, sleep quality, environment, and social relationships. Older age was correlated with lower psychological health (β = −0.054, *P* = .041). Anxiety was associated with much lower psychological health (β = −1.539, *P* = .000). Severe depression was associated with gradually lower psychological health (β = −1.144 to −2.478, *P* ≤ .014). Poor sleep was associated with lower psychological health (β = −0.096, *P* = .050), while a better environment (β = 0.105, *P* = .044) and stronger social relationships (β = 0.167, *P* = .027) improved it.

In the environmental domain, being aged 46–60 years (β = −2.01, *P* = .045) and mild depression (β = −1.197, *P* = .047) were associated with lower environmental health. By contrast, having a secondary school education (β = 2.679, *P* = .002), better physical health (β = 0.103, *P* = .028), stronger social relationships (β = 0.413, *P* = .000), and higher psychological health (β = 0.114, *P* = .045) predicted higher environmental quality. In the social relationship domain, a better environment (β = 0.199, *P* = .000), higher physical health (β = 0.191, *P* = 0.000), and higher psychological health (β = 0.087, *P* = .026) were significant and positively associated with social relationships (Table 9).

**Table 9:** Multivariable regression analysis of factors associated with WHOQOL-BREF overall QoL (*n* = 370).

Variable	Coef.	SE	t-value	*P*-value	95% CI Sig
Age	0.021	0.016	1.34	.180	−0.010, 0.051
Age group (years)					
31–45	−0.222	0.413	−0.54	.591	−1.035, 0.590
46–60	−0.473	0.571	−0.83	.408	−1.597, 0.650
≥61	−0.558	0.793	−0.70	.483	−2.118, 1.003
Time of diagnosis after the initial symptoms					
≥2 years	−0.331	0.190	−1.74	.082	−0.705, 0.043*
Educational status					
Primary school	0.485	0.199	2.44	.015	0.093, 0.877**
Secondary school and above	0.340	0.496	0.69	.494	−0.635, 1.315
Family size					
4–6	−0.302	0.309	−0.98	.330	−0.910, 0.307
≥10	−0.648	1.019	−0.64	.525	−2.651, 1.356
Level of anxiety					
Mild	−0.112	0.256	−0.44	.660	−0.615, 0.390
Moderate	−0.069	0.352	−0.20	.844	−0.763, 0.624
Severe	−0.403	0.435	−0.93	.354	−1.258, 0.452
Level of depression					
Mild	−0.650	0.341	−1.91	.057	−1.321, 0.021*
Moderate	−0.504	0.501	−1.01	.315	−1.491, 0.482
Moderately severe	−1.731	0.596	−2.91	.004	−2.902, -0.559***
BCG vaccination					
Yes	−0.088	0.222	−0.40	.691	−0.525, 0.349
PSQI global score	−0.037	0.029	−1.29	.199	−0.094, 0.020
WHO BREEF environmental domain	1.047	0.031	34.01	.000	0.986, 1.107***
WHO BREEF physical health domain	1.036	0.026	39.24	.000	0.984, 1.088***
WHO BREEF social relationships domain	1.076	0.045	24.07	.000	0.988, 1.164***
WHO BREEF psychological health domain	1.059	0.032	33.08	.000	0.996, 1.122***
Presence of visible lesions					
Yes	−0.322	0.216	−1.49	.137	−0.746, 0.102
Leprosy disability grades					
Grade 1	0.120	0.245	0.49	.625	−0.362, 0.602
Grade 2	0.018	0.218	0.08	.933	−0.411, 0.447
Attribution of leprosy					
Supernatural	0.059	0.239	0.25	.805	−0.411, 0.529
Hereditary	−0.230	0.322	−0.71	.475	−0.864, 0.404
Don’t know	−0.301	0.302	−1.00	.319	−0.894, 0.293
Changing of residence after diagnosis due to stigma					
Yes	0.063	0.221	0.28	.777	−0.372, 0.497
SARI stigma scores	0.031	0.034	0.94	.349	−0.035, 0.097
Experienced	−0.038	0.038	−1.01	.315	−0.113, 0.036
Internalized	−0.034	0.046	−0.75	.453	−0.124, 0.056
Self-stigma	−0.050	0.060	−0.84	.401	−0.168, 0.067
Alienation	0.008	0.044	0.19	.852	−0.078, 0.094
Stereotype	−0.077	0.050	−1.54	.124	−0.176, 0.021
Discrimination	−0.087	0.054	−1.62	.106	−0.193, 0.019
Social	−0.018	0.048	−0.37	.712	−0.112, 0.077
Constant	2.527	1.150	2.20	.029	0.265, 4.788**

*** *P* < .01, ** *P* < .05, * *P* < .1.

BCG-Bacillus Calmette–Guérin vaccine.

### Sleep quality

Among the 370 participants, 44 (11.9%) had a good sleep quality, 225 (60.8%) had moderate sleep disturbance, and 101 (27.3%) had severe sleep disturbance. Across all categories, a higher proportion of participants at an advanced disease stage reported poorer sleep quality. Overall, sleep disturbance was more common among participants at an advanced disease stage. Lower-income groups had moderate and severe sleep disturbances. Sleep disturbance was more common among older participants, specifically those aged ≥61 years.

### Predictors of sleep quality

A higher total stigma score was significantly associated with higher PSQI global scores (β = 0.087, *P* = .044), indicating that individuals with greater overall stigma had poorer sleep quality. Participants with primary school education had significantly higher PSQI scores compared with those without formal education (β = 0.721, *P* = .047). A higher psychological health domain score on the WHO BREF was associated with lower PSQI scores (β = –0.129, *P* = .031).

By contrast, those who had higher levels of experienced and self-stigma were both significantly associated with higher PSQI scores (β = 0.121, *P* = .022 and β = 0.202, *P* = .040, respectively), indicating poorer sleep quality among these individuals (Supplementary file 4).

### Mental health status

#### Depression

Most patients with depression were at an advanced disease stage. Among 370 patients with leprosy, 26.2% experienced depression. Depression was more prevalent among males (58.8%) and among those with G2D (47.4%). Depression was also high among married individuals (56.7%) and those from low-income groups (Supplementary file 5a-d).

#### Predictors of depressions

In the linear regression analysis, educational status, anxiety scores, and the psychological health domain of the WHOQOL-BREF were identified as predictors of depression. Higher educational status (i.e. secondary school or above) was associated with lower depressive symptoms (β = –2.294, *P* = .025). Higher anxiety scores were significantly associated with higher depressive scores (β = 0.736, *P* < .001). Better psychological health was associated with lower depressive scores (β = –0.178, *P* = .006) (Supplementary file 6).

#### Anxiety

Among the study participants, 90 (24.3%) experienced anxiety. More than two-thirds (67.8%) of those with anxiety were males. Individuals with G2D (42.2%) and G1D (41.1%) disabilities reported the highest levels of anxiety compared with those in G0D. Anxiety was highest in lower-income groups.

#### Predictors of anxiety

In the linear regression analysis, older age was significantly associated with lower anxiety levels (β = −0.029, *P* = .016). For each additional year of age, the GAD-7 score decreased by ~0.03. Higher depressive symptoms, measured by the PHQ-9 total score, were strongly associated with increased anxiety (β = 0.722, *P* < .001). For each one-point increase in the PHQ-9 total score, GAD-7 increased by 0.722 points (β = 0.722, *P* < .001). In addition, individuals who attributed leprosy as hereditary had lower anxiety scores compared with those who attributed it to supernatural causes (β = −1.561, *P* = .008) (Supplementary file 7).

## Discussion

The current study provides a comprehensive assessment of the QoL, sleep quality, mental health, and stigma among people affected by leprosy in urban rehabilitation centres in Ethiopia. The findings reveal that the burden of leprosy extends beyond its clinical manifestations for affected individuals, impacting them socially and psychologically. For individuals affected by leprosy, being at an advanced disease stage, disability grade, mental health issues, stigma, and socioeconomic conditions are factors that drive diminished overall QoL.

Sociodemographic factors also played a key role in shaping QoL. Younger individuals, those with higher education, and those with a higher income tended to have better QoL, especially in physical, psychological, and environmental areas. Education likely enhances health literacy, coping skills, and access to resources, while higher income helps to protect against financial stress. By contrast, widowed participants and those living in extreme poverty faced much poorer QoL, highlighting the combined social and economic challenges. These findings align with studies conducted in India and Brazil (leprosy-endemic settings), which showed that younger, educated, and higher-income individuals tend to report better QoL, while widowed individuals and those in extreme poverty experience poorer outcomes.^21,22^

A cross-sectional study from India, conducted among 125 people in a rehabilitation home, also found that physical, psychological, social, and environmental scores varied with marital status, employment, age, and disease duration,^23^ underscoring the impact of disability and sociodemographic status on QoL. A community-based study in Nepal among people affected by leprosy^12^ and a study in Bangladesh^24^ showed that social relationships are disproportionately affected and that stigma plays a central role in limiting social engagement, regardless of clinical severity. These patterns were found to be consistent both in communities and in the rehabilitation centres.

Several key variables were identified as major determinants of stigma, mental health, sleep quality, and overall QoL. Stigma stood out as the main factor, displaying strong correlations with elevated depression and anxiety scores, and deterioration of overall QoL, including sleep quality. This effect was notably high in the WHO BREF psychological and social relationship domains. Depression was prevalent in more than one-quarter of the studied population and emerged as a negative influencer of global QoL. Sleep disturbances were another significant issue, affecting almost 90% of participants. Poor sleep quality was more common in those at an advanced disease stage, of older age, in a lower income group, and in those with higher SARI stigma scores. The links between poor sleep, depression, and anxiety suggest a vicious cycle whereby psychological distress, stigma, and sleep issues exacerbate each other, further diminishing QoL. Stigma plays a central role, leading to anxiety, depression, poorer sleep quality, and diminished overall QoL. It also acts as a catalyst, triggering a cascade of negative effects by reinforcing negative beliefs and social isolation. Moreover, stigma prevents leprosy-affected individuals from seeking medical care, thereby worsening the cycles of psychological distress, resulting in poorer mental health outcomes (Figure 2).

**Figure 2:**
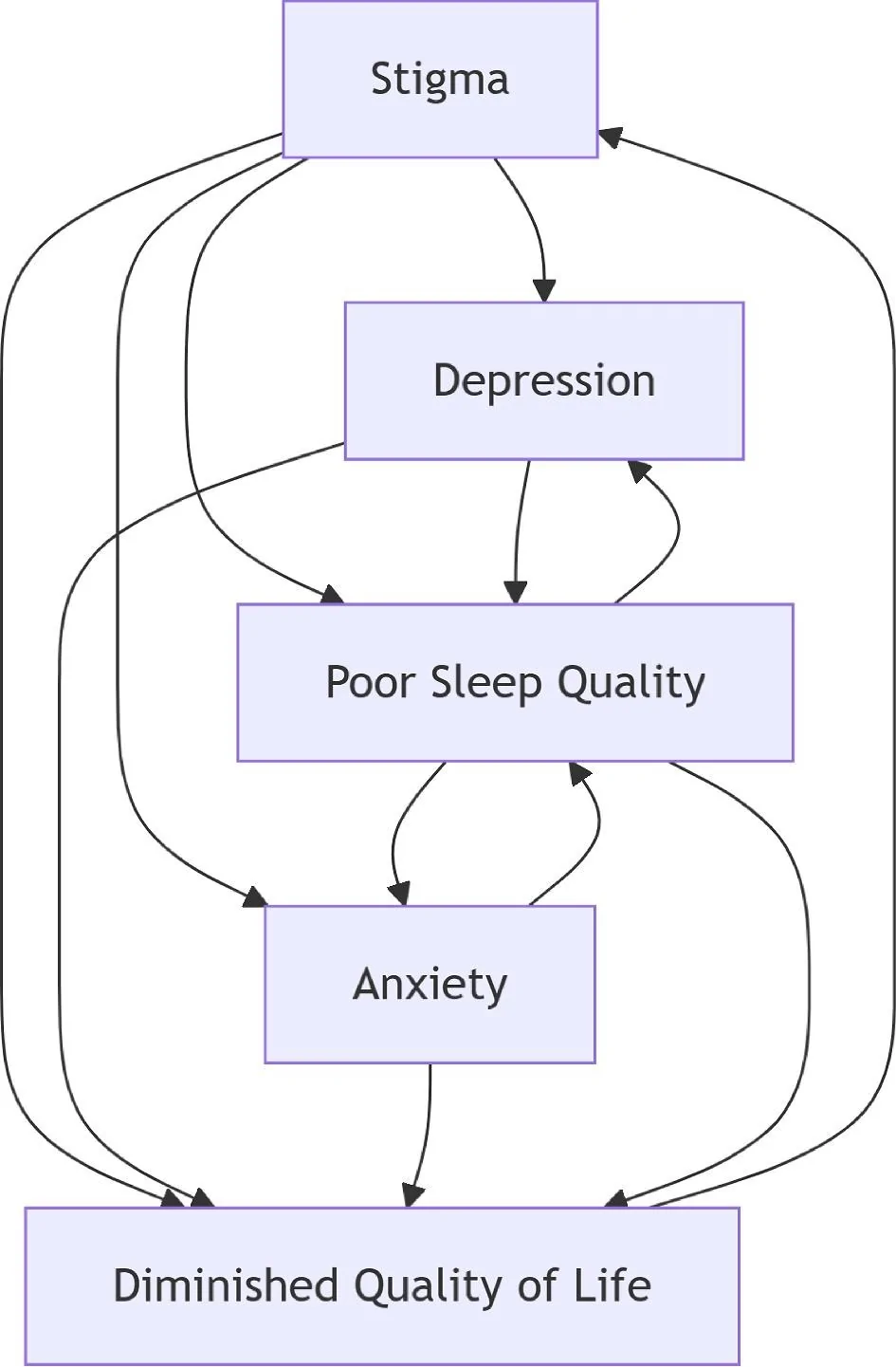
Vicious cycle of stigma as a driver of anxiety, depression and diminished QoL (n=370).

Stigma, psychological distress, and lower QoL are closely linked for people affected by leprosy. In this study, stigma had significant positive correlations with depression and anxiety, and a negative relationship with overall QoL. This finding matches evidence from Nepal, where stigma was a major factor in both depression and reduced QoL for people affected by neglected tropical diseases.^12^ A study from India reported that people affected by leprosy experience high levels of depression and anxiety. Mental health outcomes are significantly influenced by stigma, disability, and socioeconomic conditions.^25^ Recent evidence also shows that the psychological and social aspects of QoL are impacted more than the physical aspects.

Further evidence from India revealed that stigma, mental health issues, and disease severity are key factors in poor QoL, particularly impacting psychological well-being and social connections.^26^ These findings closely match our results, where social relationships and psychological health were the most affected areas of QoL.

Stigma emerged as a crucial factor influencing various outcomes. About one-quarter of participants faced stigma, with a greater burden on those at an advanced disease stage. Stigma showed a positive link with depression, anxiety, and poor sleep, while negatively impacting all QoL domains, disproportionately affecting psychological and social well-being. These results confirm that stigma is not just a social problem but a key factor affecting mental health and overall QoL in individuals affected by leprosy.

A community-based studies conducted in Nepal and Bangladesh revealed that stigma is not merely a social problem but a core determinant of mental health and diminished QoL, particularly in the psychological and social domains. These findings align with the WHO’s ‘biopsychosocial model of health’, which emphasizes that mental health and sleep quality are integral components of overall well-being and are closely intertwined with social exclusion and chronic stress in marginalized populations.^27^

Certain sociodemographic factors, such as older age, lower educational attainment, being a widower, having a smaller family size, and living in the lowest income group, were predictors of poor QoL, mental health problems, and sleep disturbances. This finding is similar to that from a multicentre community-based, cross-sectional study conducted in India, which also reported that depression and anxiety were common among people affected by leprosy, particularly among older age groups.^28^

In terms of clinical aspects, being at an advanced disease stage and the presence of comorbidities appeared to diminish QoL, with the physical and psychological domains of QoL being the most affected. Advanced disease stage and disability were common, especially among older participants and those who were widowed or married.

Many participants had visible lesions, reflecting delays in diagnosis and challenges in disease control. This finding was similar to a study conducted among individuals affected by leprosy at Bisidimo General Hospital in Ethiopia, which reported that the presence of comorbidities, delay in diagnosis, and mental health outcomes were predictors for QoL.^26^ A scoping review across multiple countries conducted in Brazil also revealed that delayed diagnosis, physical disability, and stigma were the most consistent factors associated with poor QoL among people affected by leprosy.^29^

The overall QoL was moderate, but there were significant differences across various domains. Environmental, psychological, and physical health domain scores were relatively better than those for the social relationships domain. The social relationships domain was low in both the advanced and non-advanced disease-stage groups. Consistently low scores in the social relationship domain indicate deep-rooted cultural and religious misunderstandings about leprosy.

On the other hand, our finding contradicts that reported in a cross-sectional study conducted in Brazil among leprosy-affected individuals in a rehabilitation centre that reported higher scores in the social domains of the WHO BREF QoL.^30^ This difference could be due to differences in culture, religion, social support, and healthcare context, all of which influence how leprosy affects QoL domains.

In many settings, many individuals still view leprosy as either a punishment from God, a curse, or hereditary, rather than as an infectious disease.^31^ Embedded in local cultural and religious stories, these beliefs shape how the community views those affected by leprosy and the stigma associated with it. In this study, we observed that leprosy stigma persists, no matter how severe the disease or whether skin lesions are visible. The word leprosy itself has strong negative connotations that affect not just the individuals affected by the disease, but also their families, relatives, and entire family lineages. This ‘courtesy stigma’ leads to social exclusion, discrimination, and a loss of social status.^32^ It reinforces fear, silence, and delays in seeking care, contributing to disability and ongoing transmission of disease.

Lastly, beliefs about the cause of leprosy influenced mental health outcomes. People who attributed leprosy to supernatural causes experienced higher levels of anxiety compared with those who attributed it to hereditary causes. Those who believed the disease had a hereditary origin, even although still incorrect and stigmatizing, did not imply the same level of moral judgement or existential threat. The perception of hereditary origin frames the disease as unfortunate, rather than as a spiritual punishment. This could potentially lessen the severity of feelings of shame and fear, leading to potentially lower levels of anxiety compared with beliefs in supernatural causes. This is consistent with the findings from a study conducted in Nigeria among individuals affected by leprosy, which revealed that causal beliefs remain important factors in stigma and mental health problems.^33^ Incorrect perceptions of leprosy increase the psychological burden of shame, self-blame, and fear, as those affected by leprosy may come to see themselves as cursed, excluded, and condemned by society because of perceived moral failures.

### Strengths and limitations of the study

This multisite study aims to provide an in-depth assessment of QoL, sleep quality, mental health, and stigma in individuals affected by leprosy in Ethiopia. It utilizes standardized and validated tools in the assessment. It included major rehabilitation centres to enhance internal validity. The limitations of the study include the lack of a non-leprosy control for comparison, as well as the study participants only being recruited from rehabilitation centres. Social desirability, in addition to over-reporting and under-reporting by participants from institutional settings, could have influenced QoL.

## Conclusions

This study demonstrates that leprosy profoundly affects QoL through the combined effects of physical disability, mental health problems, stigma, sleep disturbances, and socioeconomic disadvantages. Protective factors such as primary education, higher income, larger family size, and better physical and mental health were associated with improved well-being, highlighting the disease’s broader implications for human development beyond its medical dimensions. Reducing the impact of stigma through education, counselling, and social reintegration activities is fundamental to enhancing mental health and QoL. The detrimental effects of stigma can be partially mitigated by integrating mental health, psychosocial, and sleeping health interventions into leprosy rehabilitation facilities. Ultimately, reducing stigma and its impact on mental health is important, not only for improving personal well-being, but also for realizing the WHO’s vision of dignity and ending discrimination against individuals affected by leprosy.

## Supplementary Material

trag064_Supplemental_Files

## Data Availability

All relevant data are included in the manuscript and supplementary files. We have also uploaded anonymized raw data (Supplementary file 8).
