# Supplementary material for: Quality of life, stigma, and mental health, in individuals affected by leprosy: a cross-sectional study
Source: Trans R Soc Trop Med Hyg. 2026 May 28;120(10):1027–39. doi: 10.1093/trstmh/trag064 (PMC13635672; doi:10.1093/trstmh/trag064)
Supplement: Supplementary file 1 [file trag064_supplemental_files.zip › Supplementary file 1.docx]

**Supplementary file 1: Variance inflation factor (before and after corrections)**

| **Variables** | **VIF (Before Correction)** | **1/VIF (Before)** | **VIF (After Correction)** | **1/VIF (After)** |
| --- | --- | --- | --- | --- |
| Total PHQ9 score | 15.8 | 0.1 | 2.8 | 0.4 |
| Total GAD7 score | 15.3 | 0.1 | 2.7 | 0.4 |
| Anxiety category | 13.9 | 0.1 | - | - |
| Depression severity | 13.7 | 0.1 | - | - |
| Age | 10.3 | 0.1 | 1.3 | 0.8 |
| Age category | 10.1 | 0.1 | - | - |
| PSQI global score | 4.7 | 0.2 | 4.6 | 0.2 |
| PSQI category | 4.5 | 0.2 | 4.5 | 0.2 |
| WHO BREEF environmental domain | 1.5 | 0.7 | 1.4 | 0.7 |
| WHO BREEF social relationships domain | 1.4 | 0.7 | 1.4 | 0.7 |
| WHO BREEF psychological health domain | 1.4 | 0.7 | 1.3 | 0.8 |
| Presence of visible lesions | 1.3 | 0.8 | 1.3 | 0.8 |
| Leprosy grade | 1.2 | 0.8 | 1.2 | 0.9 |
| Income category | 1.1 | 0.9 | 1.1 | 0.9 |
| Perceived attributions of leprosy | 1.1 | 0.9 | 1.1 | 0.9 |
| Comorbidities | 1.1 | 0.9 | 1.0 | 1.0 |
| Changing of residence after diagnosis due to stigma | 1.1 | 0.9 | 1.1 | 1.0 |
| Family history of leprosy | 1.0 | 1.0 | 1.0 | 1.0 |
| Mean VIF | **5.6** | - | **1.9** | - |
