# Supplementary material for: Quality of life, stigma, and mental health, in individuals affected by leprosy: a cross-sectional study
Source: Trans R Soc Trop Med Hyg. 2026 May 28;120(10):1027–39. doi: 10.1093/trstmh/trag064 (PMC13635672; doi:10.1093/trstmh/trag064)
Supplement: Supplementary file 1 [file trag064_supplemental_files.zip › Supplementary file 2.docx]

Median scores

| WHO BREEF domains | Female | Male |
| --- | --- | --- |
| Environment | 17.5 | 18.07 |
| Psychological Health | 16.91 | 17.09 |
| Physical Health | 15.88 | 16.05 |
| Social Relationships | 7 | 7 |

Supplementary file 2: WHO BREEF domains across genders group (n=370)
