# Supplementary material for: Quality of life, stigma, and mental health, in individuals affected by leprosy: a cross-sectional study
Source: Trans R Soc Trop Med Hyg. 2026 May 28;120(10):1027–39. doi: 10.1093/trstmh/trag064 (PMC13635672; doi:10.1093/trstmh/trag064)
Supplement: Supplementary file 1 [file trag064_supplemental_files.zip › Supplementary file 4.docx]

**Supplementary file 4**: Multivariable linear regression analysis of factors associated with global PSQI (n=370)

| **Predictor** | **Coef.** | **Std. Err.** | **t-value** | **p-value** | **95% CI** | **Sig** |
| --- | --- | --- | --- | --- | --- | --- |
| SARI_stigma_score | -0.087 | 0.043 | -2.02 | 0.044 | -0.172, -0.002 | ** |
| **Educational status** |  |  |  |  |  |  |
| Primary school | 0.721 | 0.361 | 2.00 | 0.047 | 0.011, 1.432 | ** |
| Secondary school and above | 0.640 | 0.891 | 0.72 | 0.473 | -1.113, 2.392 |  |
| **Family size** |  |  |  |  |  |  |
| 4–6 | -0.042 | 0.367 | -0.12 | 0.908 | -0.764, 0.680 |  |
| 7–10 | -0.138 | 0.542 | -0.25 | 0.799 | -1.205, 0.929 |  |
| ≥10 | 0.344 | 1.857 | 0.19 | 0.853 | -3.308, 3.997 |  |
| **Level of anxiety** |  |  |  |  |  |  |
| Mild anxiety | -0.335 | 0.472 | -0.71 | 0.479 | -1.263, 0.594 |  |
| Moderate anxiety | -0.809 | 0.657 | -1.23 | 0.219 | -2.101, 0.483 |  |
| Severe anxiety | 0.072 | 0.808 | 0.09 | 0.929 | -1.517, 1.661 |  |
| **Depression severity** |  |  |  |  |  |  |
| Minimal | 0.470 | 0.469 | 1.00 | 0.317 | -0.453, 1.392 |  |
| Mild | 0.868 | 0.643 | 1.35 | 0.178 | -0.397, 2.133 |  |
| Moderate | 1.381 | 0.935 | 1.48 | 0.141 | -0.458, 3.220 |  |
| Moderately Severe | 1.964 | 1.116 | 1.76 | 0.079 | -0.230, 4.159 | * |
| WHO BREEF environmental domain | 0.079 | 0.055 | 1.43 | 0.153 | -0.029, 0.187 |  |
| WHO BREEF physical health | 0.066 | 0.047 | 1.39 | 0.164 | -0.027, 0.158 |  |
| WHO BREEF psychological health | -0.129 | 0.059 | -2.17 | 0.031 | -0.246, -0.012 | ** |
| **Income group** |  |  |  |  |  |  |
| Moderate Poverty | -0.410 | 0.524 | -0.78 | 0.434 | -1.442, 0.621 |  |
| Near Poor/Vulnerable | -0.437 | 0.517 | -0.85 | 0.398 | -1.453, 0.580 |  |
| Low-Income | -0.531 | 0.494 | -1.08 | 0.283 | -1.503, 0.440 |  |
| Middle-Income | -0.885 | 0.630 | -1.41 | 0.161 | -2.124, 0.353 |  |
| Upper-Income/Affluent | -0.622 | 0.836 | -0.74 | 0.457 | -2.265, 1.022 |  |
| **Leprosy disability grades** |  |  |  |  |  |  |
| Grade 1 | -0.727 | 0.438 | -1.66 | 0.098 | -1.587, 0.134 | * |
| Grade 2 | -0.151 | 0.385 | -0.39 | 0.695 | -0.908, 0.606 |  |
| **Attribution** |  |  |  |  |  |  |
| Supernatural | -0.172 | 0.446 | -0.39 | 0.699 | -1.049, 0.704 |  |
| Hereditary | -0.857 | 0.605 | -1.42 | 0.158 | -2.048, 0.334 |  |
| Don't know | 0.179 | 0.561 | 0.32 | 0.750 | -0.925, 1.282 |  |
| Experienced stigma | 0.121 | 0.053 | 2.31 | 0.022 | 0.018, 0.225 | ** |
| Internalized stigma | 0.107 | 0.067 | 1.59 | 0.113 | -0.025, 0.239 |  |
| Self-stigma | 0.202 | 0.098 | 2.07 | 0.040 | 0.010, 0.394 | ** |
| Alienation | 0.097 | 0.069 | 1.42 | 0.157 | -0.038, 0.232 |  |
| Stereotyping | 0.149 | 0.081 | 1.84 | 0.067 | -0.011, 0.309 | * |
| Discrimination | 0.127 | 0.099 | 1.28 | 0.202 | -0.068, 0.322 |  |
| Constant | 6.951 | 1.834 | 3.79 | 0.000 | 3.344, 10.558 | *** |
| *** p < 0.01, ** p < 0.05, * p < 0.1 | | | | | | |
