# Supplementary material for: Quality of life, stigma, and mental health, in individuals affected by leprosy: a cross-sectional study
Source: Trans R Soc Trop Med Hyg. 2026 May 28;120(10):1027–39. doi: 10.1093/trstmh/trag064 (PMC13635672; doi:10.1093/trstmh/trag064)
Supplement: Supplementary file 1 [file trag064_supplemental_files.zip › Supplementary file 5a-d.docx]

**Supplementary file 5 (a-d)**: Distribution of depression by socio-economic and leprosy disability grade - (n = 370)

Supplementary file 5a. Distribution of depression by gender among study participants (n=370)

| Depression | Gender | | | |
| --- | --- | --- | --- | --- |
|  | Female | Male | Total | p-value |
| No | 130 (76.5%) | 143 (71.5%) | 273 | 0.279 |
| Yes | 40 (23.5%) | 57 (28.5%) | 97 |  |
| Total | 170 (100%) | 200 (100%) | 370 |  |

Supplementary table 5b. Distribution of depression by marital status among study participants (n=370)

| **Depression** | **Marital status** | | | | | |
| --- | --- | --- | --- | --- | --- | --- |
|  | **Married** | **Separated** | **Single** | **Widow/Widower** | **Total** | **p-value** |
| No | 131 (70.4%) | 37 (78.7%) | 40 (71.4%) | 65 (80.3%) | 273 | **0.310** |
| Yes | 55 (29.6%) | 10 (21.3%) | 16 (28.6%) | 16 (19.7%) | 97 |  |
| Total | 186 (100%) | 47 (100%) | 56 (100%) | 81 (100%) | 370 |  |

Supplementary file 5c. Distribution of depression by leprosy disability grade among study participants (n=370)

|  | **Leprosy disability grade** | | | | |
| --- | --- | --- | --- | --- | --- |
| **Depression** | **Grade 0** | **Grade 1** | **Grade 2** | **Total** | **p-value** |
| No | 92 (72.4%) | 72 (81.8%) | 109 (70.3%) | 273 |  |
| Yes | 35 (27.6%) | 16 (18.2%) | 46 (29.7%) | 97 |  |
| Total | 127 (100%) | 88 (100%) | 155 (100%) | 370 | **0.134** |

Supplementary file 5d. Distribution of depression by income group among study participants (n=370)

| Depression | Income group | | | | | | | |
| --- | --- | --- | --- | --- | --- | --- | --- | --- |
|  | Extreme Poor | Moderate Poor | Near Poor | Low Income | Middle Income | Upper Income | Total | p-value |
| No | 69 (71.9%) | 42 (67.7%) | 49 (71.0%) | 62 (73.8%) | 33 (84.6%) | 18 (90.0%) | 273 | 0.243 |
| Yes | 27 (28.1%) | 20 (32.3%) | 20 (29.0%) | 22 (26.2%) | 6 (15.4%) | 2 (10.0%) | 97 |  |
| Total | 96 (100%) | 62 (100%) | 69 (100%) | 84 (100%) | 39 (100%) | 20 (100%) | 370 |  |
