# Supplementary material for: Quality of life, stigma, and mental health, in individuals affected by leprosy: a cross-sectional study
Source: Trans R Soc Trop Med Hyg. 2026 May 28;120(10):1027–39. doi: 10.1093/trstmh/trag064 (PMC13635672; doi:10.1093/trstmh/trag064)
Supplement: Supplementary file 1 [file trag064_supplemental_files.zip › Supplementary file 7.docx]

**Supplementary file 7**. Multivariable regression analysis of factors associated with anxiety (n=370)

| Predictor | β (Coef.) | SE | t | p | 95% CI | Sig |
| --- | --- | --- | --- | --- | --- | --- |
| Total stigma score | -0.010 | 0.008 | -1.21 | 0.228 | -0.026, 0.006 |  |
| Age | -0.029 | 0.012 | -2.48 | 0.014 | -0.052, -0.006 | ** |
| Sex (ref: Female) |  |  |  |  |  |  |
| Male | 0.648 | 0.371 | 1.75 | 0.082 | -0.081, 1.377 | * |
| Time of diagnosis following the initial symptoms |  |  |  |  |  |  |
| Two years and above | 0.056 | 0.389 | 0.14 | 0.885 | -0.708, 0.821 |  |
| PHQ9 score | 0.721 | 0.035 | 20.47 | <0.001 | 0.652, 0.791 | *** |
| Family history of leprosy |  |  |  |  |  |  |
| Yes | -1.951 | 1.334 | -1.46 | 0.144 | -4.575, 0.672 |  |
| PSQI global | -0.037 | 0.059 | -0.62 | 0.533 | -0.153, 0.079 |  |
| Physical health | 0.099 | 0.053 | 1.86 | 0.064 | -0.006, 0.204 | * |
| Social relationships | 0.006 | 0.086 | 0.07 | 0.942 | -0.162, 0.175 |  |
| Psychological health | -0.115 | 0.064 | -1.80 | 0.073 | -0.241, 0.011 | * |
| Income level (ref: Extreme) |  |  |  |  |  |  |
| Moderate Poverty | -0.396 | 0.564 | -0.70 | 0.483 | -1.504, 0.713 |  |
| Near Poor/Vulnerable | -0.268 | 0.559 | -0.48 | 0.633 | -1.368, 0.832 |  |
| Low-Income | 0.148 | 0.533 | 0.28 | 0.781 | -0.900, 1.196 |  |
| Middle-Income | -0.307 | 0.692 | -0.44 | 0.658 | -1.668, 1.054 |  |
| Upper-Income | 0.074 | 0.916 | 0.08 | 0.935 | -1.728, 1.876 |  |
| Leprosy disability (ref: None) |  |  |  |  |  |  |
| Grade 1 | -0.205 | 0.477 | -0.43 | 0.668 | -1.143, 0.733 |  |
| Grade 2 | 0.467 | 0.419 | 1.11 | 0.266 | -0.357, 1.292 |  |
| Attribution (ref: Other) |  |  |  |  |  |  |
| Infection | 0.230 | 0.482 | 0.48 | 0.634 | -0.718, 1.178 |  |
| Hereditary | -1.600 | 0.583 | -2.74 | 0.006 | -2.747, -0.453 | *** |
| Don’t know | -0.364 | 0.540 | -0.67 | 0.501 | -1.427, 0.698 |  |
| Changing of residence after diagnosis due to stigma |  |  |  |  |  |  |
| Yes | -0.667 | 0.446 | -1.49 | 0.136 | -1.545, 0.211 |  |
| Constant | 5.033 | 2.095 | 2.40 | 0.017 | 0.913, 9.153 | ** |
| *** p < 0.01, ** p < 0.05, * p < 0.1 | | | | | | |
